# New host and lineage diversity of avian haemosporidia in the northern Andes

**DOI:** 10.1111/eva.12176

**Published:** 2014-06-26

**Authors:** Ryan J Harrigan, Raul Sedano, Anthony C Chasar, Jaime A Chaves, Jennifer T Nguyen, Alexis Whitaker, Thomas B Smith

**Affiliations:** 1 Center for Tropical Research, Institute of the Environment and Sustainability, University of CaliforniaLos Angeles, CA, USA; 2 Escuela de Biología, Universidad Industrial de SantanderBucaramanga, Colombia; 3 Department of Biology, University of MiamiCoral Gables, FL, USA; 4 Universidad San Francisco de Quito, USFQ, Colegio de Ciencias Biológicas y Ambientales, y Extensión Galápagos, Campus CumbayáCasilla, Ecuador; 5 Department of Ecology and Evolutionary Biology, University of CaliforniaLos Angeles, CA, USA

**Keywords:** Andes, avian malaria, *Haemoproteus*, haemosporidia, hummingbirds, *Leucocytozoon*, *Plasmodium*.

## Abstract

The northern Andes, with their steep elevational and climate gradients, are home to an exceptional diversity of flora and fauna, particularly rich in avian species that have adapted to divergent ecological conditions. With this diversity comes the opportunity for parasites to exploit a wide breadth of avian hosts. However, little research has focused on examining the patterns of prevalence and lineage diversity of avian parasites in the Andes. Here, we screened a total of 428 birds from 19 species (representing nine families) and identified 133 infections of avian haemosporidia (31%), including lineages of *Plasmodium*, *Haemoproteus*, and *Leucocytozoon*. We document a higher prevalence of haemosporidia at higher elevations and lower temperatures, as well as an overall high diversity of lineages in the northern Andes, including the first sequences of haemosporidians reported in hummingbirds (31 sequences found in 11 species within the family *Trochilidae*). Double infections were distinguished using PHASE, which enables the separation of distinct parasite lineages. Results suggest that the ecological heterogeneity of the northern Andes that has given rise to a rich diversity of avian hosts may also be particularly conducive to parasite diversification and specialization.

## Introduction

The biological richness and high endemism of the tropical Andes, particularly within avian species, has long been recognized (Myers et al. 2000; Orme et al. 2005). Close to 40% of all bird families occur within the tropical Andes, and nearly as many species occur here as in the neighboring Amazon Basin, a region that is 14 times larger in area (Herzog and Kattan 2011). This high taxonomic diversity is most certainly driven in part by the heterogeneous environments and variable climates that are characteristic of the steep elevational gradients of the Andes (Guarnizo et al. 2008; Richter et al. 2009; Herzog and Kattan 2011; Kieswetter and Schneider 2013).

With heterogeneity of habitats and climate comes opportunity. Parasite species that can exploit host diversity or abundance are likely to be highly successful in an environment saturated with hosts under favorable environmental conditions for parasite life cycle development (Johnson et al. 2013; Kamiya et al. 2014; Simon et al. 2014). Previous research suggests that malarial parasites are capable of taking advantage of environmental heterogeneity via niche divergence (Sehgal et al. 2010; Cornualt et al. 2013; Lacorte et al. 2013). With high host and vector heterogeneity, a wide range of evolutionary strategies can be employed by blood parasites. Generalist parasites may be able to exploit taxonomically divergent hosts with narrow ranges, and parasite specialists may dominant single hosts with broader geographic ranges within the same region (Loiseau et al. 2011). In general, parasite communities should not be excluded from notions of ‘biodiversity’; high parasite diversity often accompanies high host diversity (Ricklefs and Fallon 2002; Hechinger and Lafferty 2005; Keesing et al. 2010; Schaer et al. 2013).

Ecological gradients have long been recognized as important in driving avian species richness and diversity in the tropical Andes (Rahbek 1995; Kattan and Franco 2004; Smith et al. 2011). A ‘general pattern’ reported suggests that there is an inverse relationship between avian species richness and elevational zones in the tropical Andes (Terborgh 1977; Rahbek 1995). While this rule has been generalized to multiple species, spatial scales, and ecological systems, recent evidence suggests that this pattern may be greatly influenced by local climate, historical processes, and source-sink population dynamics (Rahbek 1997). For instance, in avian species in the tropical Andes, the relationship between species richness and elevation appears bell-shaped, with a maximum number of species observed at ~2000 meters (Kattan and Franco 2004).

Despite the interest in ecological processes that have led to the rich avian diversity and endemism of the northern Andes, there has been little research on the diversity of parasites that occur in the region, particularly in the Andes of Ecuador (although see Jones et al. 2013), and even fewer predictions as to how patterns of parasite prevalence may differ across ecological gradients. An ideal group in which to investigate these patterns is the haemosporidia (Apicomplexa, Haemosporidia), diverse parasitic protists that infect a wide variety of hosts, including humans, other mammals, and birds. Avian haemosporidia are a particularly diverse group, comprised of three major genera; *Haemoproteus* (two major groups, one of which infects only Columbiform hosts), *Plasmodium*, and *Leucocytozoon*. Although *Plasmodium* is the only genus traditionally associated with symptoms of malaria (which are anemia-like, and caused by the parasite attacking red blood cells, Cahn and Line 1998), there is evidence to suggest that both *Haemoproteus* and *Leucocytozoon* infections can adversely affect host health and survival (Merino et al. 2000; Valkiūnas 2005; Martínez-de la Puente et al. 2010), particularly in birds that have not previously been exposed to infection (Ferrell et al. 2007).

Documenting differences in the prevalence of parasites and their lineage and host diversity in a region is a fundamental first step to understanding the drivers of disease and how host species might be impacted (Echaubard et al. 2014). To better understand these drivers, we sampled members of two avian orders and nine families in Ecuador. We hypothesize that the extreme ecological gradients of the northern Andes have led to significant differences in avian haemosporidia parasite communities. Given the theoretical and empirical evidence suggesting that avian host richness is highest at mid-elevational environments (Rahbek 1997; Kattan and Franco 2004), and assuming that with increases in host diversity comes opportunity for their parasites, we predict higher parasite prevalence at these mid-elevation altitudes. Our specific objectives were to: (i) estimate the prevalence and genetic diversity of avian haemosporidia across several ecological gradients, (ii) characterize the evolutionary diversity of South America avian haemosporidia lineages to determine the host specificity of each, and (iii) use a phylogenetic approach to understand the evolutionary and ecological forces that may have shaped the trajectories of parasite lineages. By addressing these objectives, we hope to elucidate the evolutionary forces responsible for current diversity patterns of haemosporidia in the northern Andes, as well as to identify additional impacts to an avian host community already under threat from rapid anthropogenic change.

## Materials and methods

### Sample collection

All birds were captured in the field using mist nets of various sizes at localities in Ecuador between 1999 and 2004. Individual sites were visited and sampled across multiple years, and data collected represent sums of data collected across all years (Table S1). From all captured birds, morphological measurements were taken, and blood samples were collected by venipuncture for genetic analyses and screening of avian haemosporidia. A total of 428 individuals were screened for blood parasites, across broad taxonomic categories (19 species from nine families and two orders, Table 1) and a range of ecological and elevational gradients (Table S1). Samples that were collected within 5 km of each other were considered single sites, for a total of 24 sites surveyed. Samples were collected from different sites in different field seasons, and as such we could not evaluate yearly variation in prevalence at each site. We make the assumption in our analyses that prevalence differences between habitat types and ecological gradients will exceed those observed under the same ecological conditions under different seasons or years.

**Table 1:** Host species list and 28 lineages of avian haemosporidia confirmed by sequencing. Table includes five double infections that were computationally resolved and five *Leucocytozoon* infections that were not targeted, but that were recovered via reverse primer amplification using a broad primer designed to recover *Plasmodium* and *Haemoproteus*.

			*Plasmodium*							*Haemoproteus*																*Leucocytozoon*				
Species	*N*	Prev	1	2	3	4	6	11	23	5	7	8	9	10	12	13	14	15	16	17	18	19	20	21	22	L1	L2	L3	L4	L5
Adelomyia melanogenys*	24	0.17											1	1				2												
*Basileuterus fulvicauda*	1	–	1																											
*Diglossa cyanea*	82	0.55							2							1		24				3	5	1		1	1	1	1	1
*Euphonia xanthogaster*	199	0.23																			3				1					
Eutoxeres aquila*	8					1																								
*Glyphorynchus spirurus*	18	0.06					1													1										
*Mionectes striaticollis*	26	0.19		1								2																		
*Mionectes olivaceus*	25	0.04																			1									
Phaethornis baroni*	2	–																1												
Phaethornis guy*	9	–			1	2									1		1	3												
Phaethornis malaris*	3	–						2											1											
Phaethornis striigularis*	3	–			1					1								2												
Phaethornis superciliosus*	1	–																1												
Phaethornis syrmatophorus*	9	–			1					1							1	3												
Phaethornis yaruqui*	13	0.31	1														1	2												
*Pipra erythrocephala*	1	–						1																						
Thalurania fannyi*	1	–									1																			
*Thamnophilus schistaceus*	2	–						1																						
Threnetes niger*	1	–						1																						

* Indicates hummingbirds (Order: Apodiformes); all other species listed are passerines (Order: Passeriformes).

### Laboratory methods

Whole genomic DNA was extracted from blood samples collected using a DNeasy Blood & Tissue Kit (Qiagen, Valencia, CA, USA). From these extractions, a 456-bp fragment of cytochrome oxidase subunit-b (*cyt b*) of haemosporidia parasites was amplified via PCR (GenBank accession numbers KJ661246-KJ661333). We used a nested PCR protocol and thermocycling conditions, previously reported to amplify lineages (defined here as a unique sequence of parasite mitochondrial DNA, which can differ by as little as 1 base pair, provided this sequence was recovered from both forward and reverse strands) of both *Plasmodium* sp. and *Haemoproteus* sp. within avian blood samples (see Supporting Information, Waldenström et al. 2004; Chasar et al. 2009). PCR products were separated via electrophoresis in 2% agarose gels, and products that appeared similar to parasite positive controls were purified using ExoSAP-IT (USB Corporation, Cleveland, OH, USA). Bi-directional sequencing of the purified nested-PCR products was conducted using dye terminator v3.1 fluorescent labeling in an ABI PRISM 3739*xl* (Applied Biosystems, Foster City, CA, USA). The resulting sequences were aligned using Sequencher 4.8 (Gene Codes Corporation, Ann Arbor, MI, USA); forward and reverse sequences were verified and assembled to distinguish each infection, and unique lineages were identified and matched to the three closest sequence matches in both GenBank (by nucleotide blast search) and the MalAvi Database (Bensch et al. 2009). Five *Leucocytozoon* sequences were also recovered using our primer set, although these were not a targeted group (see Supporting Information). These sequences were used as an outgroup to the other haemosporidia and included in prevalence counts by site, but were excluded from all measures of genetic diversity and phylogenetic analyses.

### Resolving haemosporidia lineages

Unresolved sequences showing double peaks in electropherograms (*n* = 5) were treated as double infections (sequences represented mitochondrial DNA) and were resolved via computational phasing using the program phase 2.1.1 (Stephens et al. 2001; Stephens and Donnelly 2003). All single-lineage sequences (*n* = 73, those containing no double peaks) of *Plasmodium* and *Haemoproteus* were input as homozygous sequences in the phase input. Double infections (*n* = 5, those containing double peaks in the mitochondrial *cyt b* sequences) were arbitrarily split into two haplotypes (representing lineages here) required to run in phase (Stephens and Donnelly 2003). All positions that contained three different bases across lineages were designated as tri-allelic SNPs as required for input. A total of 78 sequences were run using the default parameters in phase (with the exception of 10x iterations and burn-in, and 2x thinning intervals, Harrigan et al. 2008). Because we did not expect recombination in lineages of *Plasmodium* or *Haemoproteus*, all runs were performed using the MS flag, which does not allow for recombination between sequences. All phase results were scrutinized according to phase uncertainties and phase probabilities (see Results).

### Prevalence and diversity of haemosporidia

Prevalence values of all haemosporidia (including *Plasmodium*, *Haemoproteus*, and *Leucocytozoon*) were calculated for each site (*n* = 11) where greater than 10 birds were sampled to estimate avian haemosporidia prevalence within a community of hosts. For each lineage, host specificity was calculated using three estimates (Poulin et al. 2011): by the basic host specificity (corrected for missed species at locations with small sample sizes (Chao 1987)), the structural host specificity (Shannon 1948), and the phylogenetic host diversity (host phylogenies were based on mitochondrial ND2 sequences, see Supporting Information) (Faith 1992). Diversity indices were calculated using the packages *vegan* and *picante* in the R statistical framework (R Foundation for Statistical Computing 2011). To account for uneven species richness, we used the ses.pd function (Webb et al. 2008) within *picante* to test each phylogenetic host diversity measured against random permutations of host phylogenies to determine whether each lineage infected significantly more or less hosts than expected against null permutations of host phylogenies.

From the lineages recovered, we performed an Analysis of Molecular Variance (amova) (Weir and Cockerham 1984), calculated using arlequin v3.5 (Excoffier and Lisher 2010) to examine the genetic variation of haemosporidia among multiple categories. First, we used nonparametric permutation methods (Excoffier et al. 1992) and treated sites as groups to test the overall pathogen genetic subdivision in Ecuador. We then grouped haplotypes according to either basic geography (either ‘East’ or ‘West’ Andes, defined as occurring on either the eastern or western slope and foothills of the Andean range), ecological gradients determined by regression analyses (see below), or by phylogeny (belonging to either Apodiformes or Passeriformes, the two avian orders we screened, and by sister-species groupings) to test whether significant pathogen genetic variation occurred within these subdivisions. The maximized index of differentiation among groups, analogous to an *F*-statistic (Wright 1965), was estimated using a distance matrix that was previously generated in PAUP (Swofford 2002) using a GTR nucleotide substitution model (Tavare 1986). The significance of population differentiation was calculated from the distribution of individual haemosporidian lineages generated from 10 000 random permutations using a pairwise GTR-corrected distance matrix.

Finally, to examine whether we had exhaustively screened for parasite diversity, we estimated diversity of haemosporidia by constructing rarefaction curves and 95% confidence intervals of parasite species richness using EstimateS v 9.1.0 (Colwell 2013), and extrapolated curves for sampling of host species beyond the 19 species sampled here. Rarefaction analyses have often been used to determine species richness curve estimates, given incomplete sampling of species or individuals, but we adapt estimates here to determine whether lineages (‘species’) were missed at sites, given our sampling of host species.

### Phylogenetic inference

To determine the relationship of recovered South American haemosporidia lineages to global haemosporidia, an ultrametric tree for parasite *cyt b* sequences was inferred using a Bayesian approach under a uniform rate, using a GTR nucleotide substitution model and gamma distributed rates (eight categories) in BEAST (Drummond et al. 2012). This approach for reconstructing haemosporidian phylogeny (Ricklefs and Outlaw 2010) included a data set of 129 operational taxonomical units (OTU) used in other taxonomic studies of avian haemosporidia (Chasar et al. 2009; Ricklefs and Outlaw 2010), including the four closest sequence matches in GenBank (Dennis et al. 2005) to each of our 23 unique lineages (excluding our five *Leucocytozoon* lineages used as an outgroup). We unlinked rate heterogeneity, base frequencies, and substitution models by codon, and ran 100 million generations across independent runs to acquire Bayesian parameter estimates from five independent chains. We used Tracer v1.5 (Drummond and Rambaut 2007) to visually inspect chain convergence to ensure parameters meet effective sample size values (>200). All runs ended with accepting rates of parameter estimates over 20% and less than 30%. The postburn-in information from convergent runs was combined to further estimate posterior distribution of topologies, as well as the maximum credibility tree.

### Variation along ecological gradients

We examined the overall haemosporidia prevalence at sites categorized by different ecological conditions (which included 19 climate variables measuring temperature and precipitation, as well as elevation and geographic coordinates; see Supporting Information) and ran several analyses to determine whether ecological variation across sites explained differences in prevalence, genetic diversity, and/or phylogeny. First, because we had no *a priori* assumption as to which gradients would be most important in explaining variation in parasite prevalence, we ran tree regressions as implemented in the *tree* package (Ripley 1996) and random forests as implemented in *randomForest* (Liaw and Wiener 2002), both operating within the R statistical framework (R Foundation for Statistical Computing 2011), to determine the top ecological variables in terms of explaining variation in parasite prevalence (See Supporting Information). Second, we used the top explanatory variables identified in these regression models when testing the amova analyses comparing the influence of ecological factors in determining the genetic diversity of haemosporidia lineages across sites (see above). Finally, we tested the topology of a maximum likelihood tree of only sequences recovered at our sites using the program GenGIS (Parks et al. 2009) to identify the optimal linear gradient angle (the angle resulting in the fewest number of crossings, or incongruences, between phylogeny and geography) for our maximum likelihood trees of *Haemoproteus* and *Plasmodium* (Parks et al. 2009).

## Results

### Resolving double infections

Double infections (*n* = 5) analyzed using the phase program yielded four perfectly resolved individuals (meaning no phase uncertainties and phase probabilities = 1.0). The remaining double infection (occurring in a *Glyphorynchus spirurus* host) showed perfect reconstructed lineages except at five base positions, where phase probabilities ranged from 0.52–0.91. Three of these sites remained ambiguous (phase probabilities <0.6), due to the fact that they represent unique base pair changes not present in the rest of the data set (Harrigan et al. 2008). Regardless of the base pair combination at these three sites, this infection represented a mixed infection of one *Plasmodium* and one *Haemoproteus* infection within the same host (Table S4). Among five double infections found, we observed this mixed infection phenomenon three times in three different host species (two within the *Phaethornis* genus).

### Prevalence and diversity of Haemosporidia

Of the 428 individual birds screened, we found a total of 133 positive samples for an overall prevalence of 31% (reported here as percent infected individuals divided by total individuals screened). Prevalence varied across sites, from a high of 60% at a site in the western Andes, to a low of 7.7% at a site in the eastern Andes (Table S1). Although small sample sizes prevented prevalence to be determined individually for *Plasmodium* and *Haemoproteus* at each site, we recovered lower numbers of *Plasmodium* infections (*n* = 14) and lineages (*n* = 7) compared with *Haemoproteus* infections (*n* = 69) and lineages (*n* = 16). Prevalence also varied between species sampled, with Passeriformes spanning the range in prevalence variation (high in *D. cyanea*: 55%, low in *M. olivaceus*: 4%, Table 1).

Reported here for the first time, we found avian haemosporidian infections in hummingbirds across a broad range of taxa within the *Trochilidae* family (Table 1). A total of 11 species of hummingbirds were found to harbor either *Plasmodium* or *Haemoproteus* infections (or both), and prevalence was relatively high in at least one of these species (*P. yaruqui*: 31%). Although sample sizes were too low to calculate prevalence values for other species, infection rates appear to support this trend of high parasite prevalence (*P. guy*: 66% *n* = 9, *P. malaris*: 100%, *n* = 3, *P. striigularis*: 100%, *n* = 3, including one double infection of both *Plasmodium* and *Haemoproteus*).

Richness and diversity of haemosporidia sampled in Ecuador consistently increased with number of species sampled (Fig. S1). The extrapolation of richness to the total number of sampled species (those screened in the dataset) estimated that between 20 and 36 lineages should be recovered, given our sampling effort (Fig. S1). However, with increasing host species sampled, estimates of new lineages recovered increase as well. Rarefaction extrapolations suggest that over 100 lineages could be present in our study area (mean estimate = 67), provided at least 100 host species were sampled.

Among the 28 unique lineages sequenced from our sampling efforts, lineages ranged from rare (i.e., occurring only once in a single individual, as in lineage SA2, SA6, or SA17) to common (appearing in multiple individuals across many taxa, as in lineages SA15 and SA11) (Table 1). Twenty of the 28 lineages recovered occurred in only a single host (consequently with a Basic Host Specificity = 1), and five of these occurred in multiple individuals. Of the remaining eight lineages that infected multiple hosts, four belonged to each of the *Plasmodium* and *Haemoproteus* groups, respectively (Table S2). These lineages infecting multiple hosts (and therefore more generalist than single-host parasites) infected different hosts with similar frequency (no significant differences were observed in Structural Host Specificity) but varied in the extent to which the specialized on phylogenetically related hosts (Table S2). Three of these lineages (1 *Plasmodium*, 2 *Haemoproteus*) infected significantly less phylogenetically diverse hosts (represented by low Phylogenetic Host Diversity scores, Table S2, Fig. S4) than expected by chance, suggesting that despite infecting multiple hosts, these parasites have specialized on a taxonomic group. In two of these three cases, the ‘host-canalized’ parasites infected only members of the *Phaethornis* genus (SA3 and SA14, Table 1, S2).

### Phylogenetic inference

**Figure 1:**
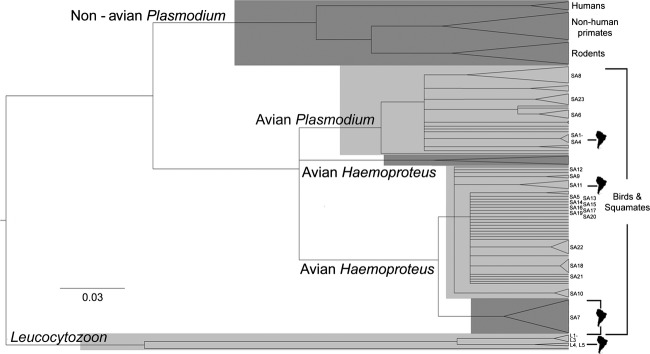
Ultrametric tree reconstructed using Bayesian inference from 129 lineages of avian haemosporidia representing global distributions. To remove ambiguity, all branches that had less than 0.95 posterior probabilities were collapsed. Lineages identified in this study are represented by labels to the right (staggered for clarity only), and monophyletic South American clades are identified with South America continent symbols. *Leucocytozoons* identified in this study were used as the outgroup to all *Plasmodium* and *Haemoproteus* lineages.

Our phylogeny of haemosporidia *cyt b* sequences (Fig. 1) clearly separated clades (1.0 posterior probability) representing avian and squamate *Plasmodium* and *Haemoproteus* lineages (2 *Haemoproteus* groups were recovered, 1 occurring only in Columbiformes) after first branching off from nonavian *Plasmodium* (lineages primarily infecting primate and rodent hosts). We found statistical support for differences in genetic diversity between *Plasmodium* and *Haemoproteus* [amova, *F*_st (GTR-corrected) 1,21_) = 0.579, Nonparametric 10, 100 permutations procedure *P* < 0.05], suggesting broad diversification in each of these groups within the northern Andes.

The avian haemosporidia sequenced in our study do not form a monophyletic group, but instead are widely dispersed within both the *Plasmodium* and *Haemoproteus* clades (Fig. 1). Despite this sequence divergence even with a relatively small study area within South America, several clades were recovered that represent monophyletic groups of haemosporidia that occur only within South America, while other monophyletic groups do not appear to have strong geographic associations.

Unique lineages of haemosporidia (*n* = 28) were found as individual or mixed infections in 83 individual hosts that resulted from nested-PCR positives. We found statistically significant genetic differentiation between 21 sites (sites with at least one positive haemosporidia sequence) across Ecuador (Table 2) [amova, *F*_st (GTR-corrected) 19,68_) = 0.1561, Exact Test of individuals distribution *P* = 0.00001]. Differentiation among sites in both sides the Andean ranges was also supported [amova, *F*_st (GTR-corrected) 1,86_) = 0.067, Exact Test of individuals distribution *P* = 0.006]. The genetic differentiation between closely related and nonclosely related groups was statistically supported in two ways. Differentiation of parasites infecting Apodiformes and Passeriformes in our data set was supported by statistical analysis [amova, *F*_st (GTR-corrected)1,85_) = 0.0343, Exact Test of individuals distribution *P* = 0.002]. Additionally, differentiation of sister-species pairs used as three individual populations showed significant support [amova, *F*_st (GTR-corrected) 2,17_) = 0.176, Exact Test of individuals distribution *P* = 0.008] (Table 2).

**Table 2:** Analysis of molecular variance (amova) of haemosporidian *cyt b* sequences found in 19 bird species of the Andes in Ecuador.

Source of variation	*N*	Number of haplotypes	Genetic diversity (EH)	*F*-statistic (%)	*P*
Geographic based comparisons					
Among sites	21	19/68	0.77 (SD 0.29)	15.61	0.000
East versus West of the Andes	2	15/17	0.79 (SD 0.14)	6.70	0.006
Elevation	2	17/14	0.74 (SD 0.11)	6.00	0.000
Temperature	2	17/14	0.73 (SD 0.12)	4.36	0.000
Phylogenetic based comparisons					
Apodiformes v. Passeriformes	2	12/19	0.79 (SD 0.03)	2.91	0.002
Sister-species	3	3/6/3	0.82 (SD 0.01)	17.60	0.008

### Variation along ecological gradients

**Figure 2:**
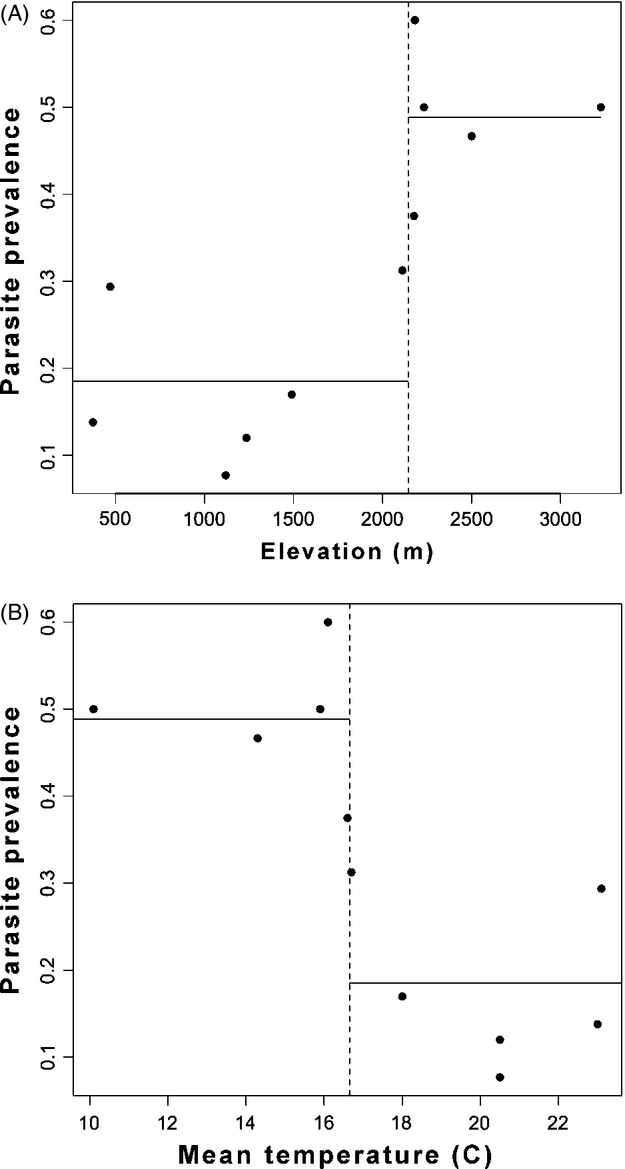
Relationship between elevation and temperature and prevalence in avian haemosporidia within the northern Andes. These two variables ranked among the most important in explaining prevalence variation under tree regression and random forest models (Fig. S2). Higher elevation sites (with lower mean annual temperatures) were found to have higher prevalences of avian haemosporidia. Vertical line represents the calculated bifurcation that best split sites according to tree regression, and horizontal lines represent prevalence means for each of those groups.

Results from regression analyses found that measures of elevation and mean annual temperature were among the top ecological predictors of haemosporidia prevalence across study sites (Figs. 2, S2). Higher elevation sites [cutoff value = 2145 meters above sea level] were found to have higher prevalence than those sites at lower elevation. These sites correspond to lower temperatures as well; sites with lower temperatures harbored higher prevalence of avian haemosporidia (<16.5°C, Fig. 2). Random forest models including just these two variables were able to explain 68% of the variation in prevalence across sites, and elevation alone (the top variable when all predictors were included) explained 63% of total variation in avian haemosporidia prevalence (only a small increase in variance explained is due to the strong inverse relationship between temperature and elevation variables, *R*^2^ = 0.97).

**Figure 3:**
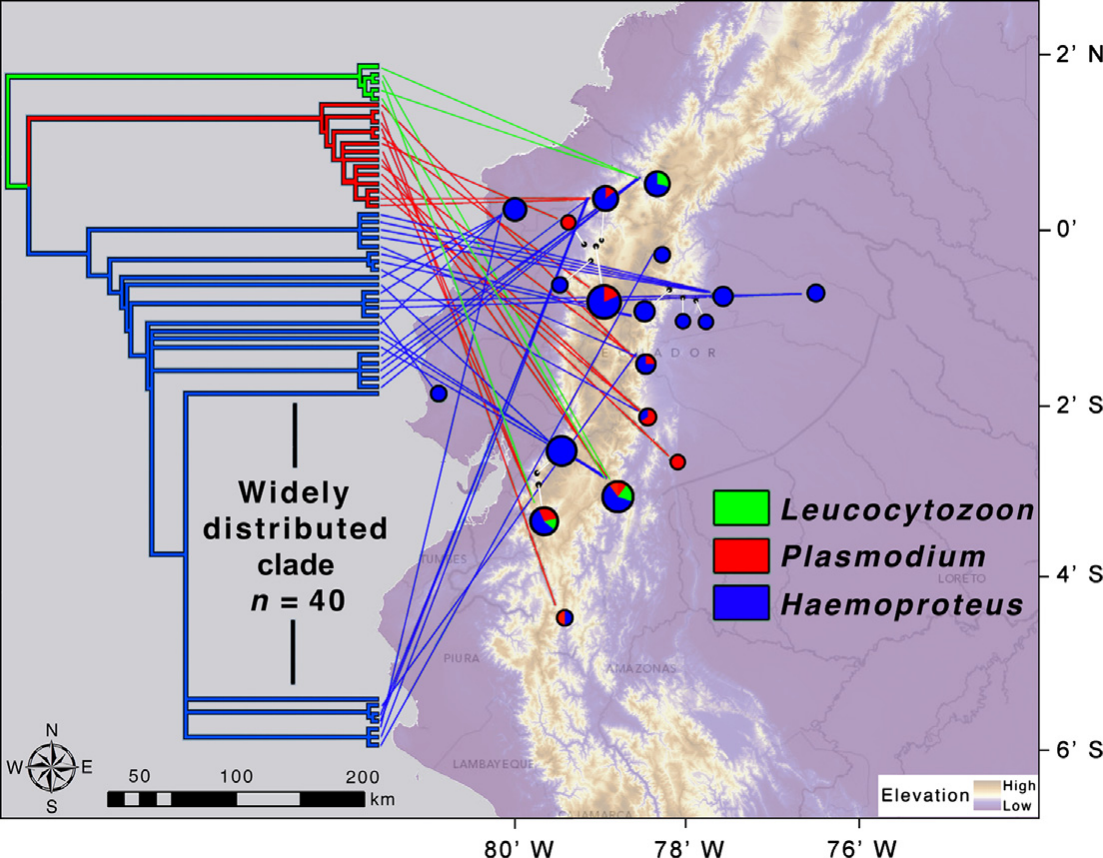
Regional distribution of *Plasmodium* and *Haemoproteus* lineages within 20 sites in Ecuador. No significant structure was observed along a latitudinal gradient, while genetic and phylogeographic differences were observed between east-west lineages and between populations at different elevations. Pie charts are proportional to the number of samples collected at each site. Four sites from the full data set (Table S1) are not included in this analyses as they either had no positive haemosporidia lineages (Jatun Sacha, Patacocha, Rio Chalpi) or had a positive for which sequence data was not acquired (Utuana).

We found significant genetic differences between low elevation and high elevation [amova, *F*_st (GTR-corrected)1,81_) = 0.0600, Exact Test of individuals distribution *P* = 0.00014] and low and high temperature [amova, *F*_st (GTR-corrected) 19,68_) = 0.0436, Exact Test of individuals distribution *P* = 0.00027] sites, broadly suggesting that parasite diversity and community structure are different within these groups across ecological gradients. The phylogeographic relationships of only avian haemosporidian lineages recovered from our study were used to examine how lineages were geographically distributed within our study region (Fig. 3). We found lineages that infect multiple hosts (SA15, SA11) dispersed across wide geographical areas, whereas other lineages were restricted to either one or two locations (for instance, SA19 found only at Cerro Bosco, and SA20 found only at Bellavista and Guandera). With the exception of one infection found at a low elevation site (Maizal, Table 1) on the eastern side of the Andes, lineages of *Plasmodium* were recovered only from sites with elevation greater than 1000 meters above sea level. While longitude was not determined to be a variable of particular importance in tree regression and random forest analyses (Fig. S2), tree topologies for both *Plasmodium* and *Haemoproteus* trees were found to be aligned across Andean ranges at optimal angles (*Plasmodium* optimal angle = 257.7°, *Haemoproteus* topology optimal angle = 101.3°) that were consistent with east-west branching (and correlated to both elevation and temperature variation) as compared to, for instance, diversification form the north Andes to the south or vice versa (Fig. S3).

## Discussion

Previous reports of malaria-like infections in hummingbirds were published more than 70 years ago (Coatney and West 1938; White et al. 1979), and more recently confirmed using current microscopy methods (Valkiūnas et al. 2004) or sporadically found (Merino et al. 2008; reviewed in Godoy et al. 2013) within single species within the *Trochilidae* family. For the first time here, we document infections of avian blood parasites (both *Plasmodium* and *Haemoproteus*) in a broad diversity of hummingbird species, as well as report wide variation in prevalence and diversity of avian haemosporidia across these and other hosts within the northern Andes. We found high prevalence of haemosporidia in one hummingbird species (31%, *P. yaruqui*), and numerous parasite lineages in this family (*n* = 12, Trochilidae), suggesting that these hosts harbor chronic infections, and that they survive infection of these endemic parasites. However, high prevalence of anemia-inducing parasites may be particularly debilitating in high-altitude species such as hummingbirds, who are likely at their ecological extremes and already under tight metabolic constraints given their body size and altitudinal range. With changes in habitat, floristic composition, and competition that are likely to result from anthropogenic changes in climate (Buermann et al. 2011), we suggest adding how haemosporidia prevalence and diversity may affect hosts as a factor in conservation of these high-altitude species.

Evidence from our analyses of ecological gradients indicates that elevation was the best predictor of haemosporidia across sites (followed by mean annual temperature, Fig. 2). We do not realistically expect that parasites or vectors are responding to elevation characteristics *per se* (for instance by taking advantage of variations in oxygen content at elevation); we suggest instead that different elevations most accurately reflect changes in biological processes and species compositions that lead to variation in haemosporidia prevalence and diversity. While a ‘general pattern’ had previously been reported suggesting an inverse relationship between avian species richness and elevation, we found that higher elevation sites (greater than 2145 m) harbor a larger percentage of infected individuals (48%), a finding that is consistent with prevalence estimates previously reported from the Andes (Jones et al. 2013), but opposite from the trends found in Australian gradients (Zamora-Vilchis et al. 2012). Higher elevations may provide habitats that may be particularly conducive to biting midges (Kaufmann et al. 2012), the primary vector of avian *Haemoproteus*, and these infections are driving high prevalence at these altitudes (Jones et al. 2013). However, we also found lineages of *Plasmodium* at these mid- and high-elevational sites, with only one infection recovered from sites sampled less than 1000 meters above sea level. Our highest elevation site, at 3227 meters above sea level, harbored only infections of *Haemoproteus* and *Leucocytozoon*, suggesting that either mosquitoes, the primary vectors of *Plasmodium*, have distributions covering the mid to high elevations (up to ~3000 m) across our study region, or that infected hosts (for instance, members of the *Phaethornis* genus, Hobson et al. 2003) are migrating across elevational gradients and carrying with them parasite lineages. This also suggests that mid-elevation habitats harbor the highest levels of parasite diversity, and along with previous findings of high diversity of hosts at these elevational ranges (Kattan and Franco 2004), suggests that mid-elevation sites may contain more biodiversity in general. While several *Plasmodium* lineages were recovered, individual lineages were found to infect a number of hosts, suggesting that *Plasmodium* in the Andes acts as more of a generalist parasite as compared to lineages of *Haemoproteus* (Table S2). The results confirm previous findings that *Leucocytozoon* is well established at higher altitudes (Haas et al. 2012). As our sampling sizes and number of sites were limited at the highest elevations, further investigation into these environments would greatly aid to support the pattern seen between altitude and host species richness in the tropical Andes (Kattan and Franco 2004), namely a decrease in species numbers at extreme altitudes (>3000 m). At elevation, although mean temperatures are lower, precipitation generally increases and could account for the nonlinear relationship recently reported between species diversity and elevation (Rahbek 1997; Kattan and Franco 2004).

Overall, the prevalence of avian haemosporidia in our study (31%) represents a higher prevalence compared with other studies in Central and South America that found overall low infection rates and haemosporidia diversity (12.8%, Bennett et al. 1991, 8; %, Valkiūnas et al. 2003; 11%, Mijares et al. 2012). However, recent work conducted in both the highlands and the tropical forests of South America suggest that prevalence may be much higher in these habitats than previously reported (Rodríguez et al. 2009; Jones et al. 2013; Lacorte et al. 2013; Svensson-Coelho et al. 2013); improved screening incorporating molecular methods may at least partially explain these discrepancies.

What other factors could contribute to these higher prevalence estimates? Transitions between habitats have been found to facilitate high genetic diversity and rates of speciation in the Andes (Thomassen et al. 2010) and elsewhere (Smith et al. 1997, 2011; Sehgal et al. 2010). Results indicating that phylogenies of both *Plasmodium* and *Haemoproteus* groups within our study region diversified across longitudinal axes, which also represent sharp elevational and temperature changes, lend further support to the idea that these transitions facilitate diversification. Interestingly, variation in avian haemosporidia diversity across elevational zones in the northern Andes may represent another selective force promoting diversity in avian hosts and/or vectors across ecological gradients, despite the fact that these types of forces are rarely considered in studies of the evolutionary drivers of diversification.

In the context of the global phylogeny of avian haemosporidia, the relationships recovered *between* and *within* clades of mammalian and avian-reptilian parasites match well the phylogenies documented elsewhere (Outlaw and Ricklefs 2010; Ricklefs and Outlaw 2010) and suggest that our recovered tree topology represents the diversity of avian haemosporidia. We found that Andean lineages were well dispersed throughout the *Plasmodium* and *Haemoproteus* clades (Fig. 1). We found no evidence to suggest that levels of genetic diversity were different between lineages of *Plasmodium* and those of *Haemoproteus* and found multiple lineages of each across sites and elevations, suggesting that sites and their parasite communities have not recently experienced periods of isolation from other sites and the parasites they harbor. This mixing of lineages across sites and regions could results from a number of evolutionary processes (or combination of them), including: (i) continual introductions of new lineages to the Andes via long-distant migrants, (ii) multiple historic introductions followed by some local adaptation and lineage formation, or (iii) smaller scale migratory movements along elevational gradients that increase local lineage diversities. Previous evidence (Hobson et al. 2003) and results from our study suggest that regional and elevational migration of at least some host species may account for parasite movement across habitats. For instance, two hummingbird species found across multiple sites and elevational zones (*P. syrmatophorus* and *A. melanogenys*) were found to harbor infections of a variety of avian *Plasmodium* and *Haemoproteus*, including the most common lineage found to infect hosts (SA15).

While the lineage diversity revealed in the northern Andes was previously unknown, rarefaction analyses suggest that additional lineages remain to be identified in this area, providing impetus for further screening of avian hosts for haemosporidia. Recent investigations in understudied tropical forests worldwide, including Australia and Papua New Guinea (Beadell et al. 2004, 2009), Africa (Loiseau et al. 2011), and the Amazon (Lacorte et al. 2013), have revealed dramatic lineage variation within the haemosporidia, and it is likely that parasite communities in the Andes are similar in their diversity and complexity. Despite this range of diversity, we also recovered several monophyletic clades found only within South America. This included one well-supported group (within *Plasmodium*) that contained only sequences recovered in this study. We found a range of specialist and generalist lineages across our study region, suggesting different evolutionary strategies being used by different lineages across elevational gradients, but caution should be taken in initial interpretation. For instance, lineage SA18, found in two Passeriformes hosts, occurred only at lowland sites, whereas SA15 was found ubiquitously throughout the region in multiple hosts (Fig. 3). While what appeared to initially be a generalist parasite (SA15 infected 40 individuals representing eight species), phylogenetic comparisons of infected hosts suggest that this lineage infects more closely related hosts than expected by chance, and exemplifies the problems associated with using only measures of host number in determining parasite generalists or specialists (Poulin et al. 2011; Svensson-Coelho et al. 2013).

Double infections of avian haemosporidia have often been excluded from analyses (Pagenkopp et al. 2008; Dimitrov et al. 2010) or separated via costly and time-intensive laboratory techniques (Beadell et al. 2004; Merino et al. 2008; Chasar et al. 2009). To our knowledge, this is the first time computational means have been used to successfully separate these double infections so that they can be included in lineage identification and measures of genetic diversity, at a fraction of the time and cost normally associated with resolving these infections. Given the fact that double infections are thought to be underrepresented (Valkiūnas et al. 2009) using traditional PCR-based screening, and their potentially additive detrimental effects on hosts (Marzal et al. 2008), we propose that careful primer selection coupled with computational resolution of lineages could greatly increase the accuracy of reported double infection prevalence, the resolution of individual haemosporidia lineages, and their effects on wildlife hosts. The fact that computational methods were able to separate infections of *Plasmodium* and *Haemoproteus* (representing large sequence divergence within *cyt b*) in all but one infection within our data set suggests the utility of this method for resolving double infection both within and between large monophyletic clades of avian haemosporidia.

We recovered sequences of avian haemosporidia from 19 species of birds representing nine families; however, caution should be taken in describing these species as hosts. It is possible that sequences of sporozoites can be recovered from avian blood despite the fact that no infection has been established (Valkiūnas et al. 2009). While microscopy should be used when possible to confirm these individuals as hosts (Valkiunas et al. 2006), there is evidence to suggest that many of the species we have recovered parasite sequences from are in fact hosts. First, we recovered multiple positives from the majority of species sampled (except for those where only a single individual was sampled), suggesting infections were not sporadic in nature. Second, sequences were often identical or closely related to other lineages found to infect hosts in South America (Table S3). Finally, as the majority of our infections were of *Plasmodium* and *Haemoproteus*, rather than of *Leucocytozoon* [where amplification of sporozoites has been identified as most problematic (Valkiūnas et al. 2009)], we are confident that the majority of our identified lineages represent circulating infections within avian hosts.

A missing component of our work is the matching, equivalently detailed analyses of vectors in this region. Various groups of avian haemosporidia can be transmitted by different primary vectors, each having unique habitat, climate, and host requirements, and understanding the evolutionary dynamics between pathogen, vector, and host components of a disease cycle is essential (Vander Wal et al. 2014). Elevational migration from hosts may be capable of transmitting avian haemosporidia great distances to new ecological habitats, but only provided the vectors necessary to complete the parasite life cycle are present. A better understanding of vector abundance and diversity will be an important next step in the understanding of the evolution and distribution of avian haemosporidia in the northern Andes.

## Data Sharing/Archiving

Data for this study are available in Genbank (Accession numbers KJ661246-KJ661333) and in the Dryad Digital Repository: http://dx.doi.org/10.5061/dryad.862h5

## Supporting Information

**Appendix S1.** Methods and results. **Figure S1.** Rarefication curve showing the increase in number of discovered lineages of avian haemosporidia as number of species sampled (at random) increase. **Figure S2.** Variable importance scores for all ecological variables and including geographic variable (latitude and longitude) used as predictors to explain avian haemosporidia prevalence in random forest models. **Figure S3.** Optimal angles of *Haemoproteus* (blue) and *Plasmodium* (red) lineages along linear axes with respect to reconstructed maximum likelihood trees. **Figure S4.** Phylogeny of host species found to be infected with avian haemosporidia in Ecuador. **Table S1.** Site locations, prevalence, and number of lineages of avian haemosporidia at sites within the study region. **Table S2.** Diversity indices of lineages of avian haemosporidia in the study region. **Table S3.** Lineages recovered and closest matches in GenBank and MalAviDatabase. **Table S4.** Double infection resolved using phase software.
