# Supplementary material for: New host and lineage diversity of avian haemosporidia in the northern Andes
Source: Evol Appl. 2014 Jun 26;7(7):799–811. doi: 10.1111/eva.12176 (PMC4227860; doi:10.1111/eva.12176)
Supplement: Supplementary file 1 — Appendix S1. Methods and results. Figure S1. Rarefication curve showing the increase in number of discovered lineages of avian haemosporidia as number of species sampled (at random) increase. Figure S2. Variable importance scores for all ecological variables and including geographic variable (latitude and longitude) used as predictors to explain avian haemosporidia prevalence in random forest models. Figure S3. Optimal angles of Haemoproteus (blue) and Plasmodium (red) lineages along linear axes with respect to reconstructed maximum likelihood trees. Figure S4. Phylogeny of host species found to be infected with avian haemosporidia in Ecuador. Table S1. Site locations, prevalence, and number of lineages of avian haemosporidia at sites within the study region. Table S2. Diversity indices of lineages of avian haemosporidia in the study region. Table S3. Lineages recovered and closest matches in GenBank and MalAviDatabase. Table S4. Double infection resolved using phase software. [file eva0007-0799-sd1.doc]

**Supporting Information**

**New Host and Lineage Diversity of Avian Haemosporidia in the Northern Andes**

Ryan J. Harrigan, Raul Sedano, Anthony C. Chasar, Jaime A. Chaves, Jennifer T. Nguyen, Alexis Whitaker, and Thomas B. Smith

**List of Contents**

Supporting Information on Methods and Results

Supplementary Figs S1 through S4

Supplementary Tables S1 through S4

**Supporting Information on Methods and Results**

**Laboratory Methods**

Parasite DNA was extracted from whole blood using the DNeasy kit (Qiagen®, Valencia, California). As samples were collected, DNA was extracted in as timely as a fashion as possible (all extractions were performed within two years of original collection date). All extractions samples were kept in -80 freezers and processed on two separate occasions; the first was performed in July of 2011, and the second in August of 2013. The earliest of these screenings occurred nearly seven years after the newest samples were collected, and we saw no degradation in the quality of DNA extracted. We could only measure total DNA extracted, not parasite DNA exclusively, but we have no reason to suspect that parasite mitochondrial DNA degrades to a greater extent than host DNA. We know of no study that reports older blood sample less likely to test positive for malaria as they age, if preserved under proper conditions, and several studies suggest that, at a variety of temperatures, no loss of detection ability is observed in blood DNA samples (Chaorattanakawee *et al*. 2003, Richardson *et al*. 2006). It remains possible that recovery of sequences is influenced by age of extraction, however a large proportion of our of recovered sequences (27/83) were collected in either 1999 or 2000, suggesting sequences of malaria mitochondrial DNA ~450bp in length can be recovered from extractions performed nearly a decade old as long as samples are stored correctly.

Blood samples were screened for *Plasmodium* and *Haemoproteus* using polymerase chain reaction (PCR). A nested PCR was used to amplify a fragment of cytochrome oxidase subunit-*b* gene (515 bp) with the primers HAEMF/HAEMR2 – HAEMNF/HAEMNR2 using the methods described in Waldenstrom *et al.* (2004). This gene is commonly used for diagnostics of these parasites (Valkiūna*s et a*l., 2009). The following primers were used. Amplifications were run using an initial denaturation at 94 ºC for 3 min, followed by 35 cycles of 95 ºC for 50 s, 53 ºC annealing for 50 s, and 72 ºC extension for 60 s, and then a final extension at 72 ºC for 5 min. This method consists of 2 parts, an initial 20 cycles of PCR that amplify a 580-bp fragment and a final 35 cycles of PCR with internally nested primers that amplify a 524-bp fragment. The initial PCR, with primers HAEMNF and HAEMNR2, was carried out in a 25-μl-volume including reagent, with the number of cycles limited to 20. For the final PCR, 1.0-μl of the PCR products from the initial PCR was used as template in a 25-μl-volume reaction with the primers HAEMF and HAEMR2. The reaction included the same reagents, in the same proportions, as the initial PCR. Also, the thermal profile was identical, except extended for 35 cycles instead of 20 cycles. After cleanup of sequence ends and removal of basepairs not recovered on all sequences, our final product was 456 base pairs in length.

We recovered five *Leucocytozoon* sequences using the above primers, but obtained only reverse sequences for these lineages, suggesting the reverse primers was complimentary to base pairs in these lineages while forward primers (either forward primer in the nested pair) were different enough to prevent annealing. These lineages were clean, unambiguous peaks across all 456 base pairs, and were positively identified as *Leucocytozoon* through comparisons in GenBank and the MalAvi Database (Table S3). These lineages were used in estimates of prevalence, and as outgroups in phylogenetic trees, but were excluded from genetic diversity estimates.

**Resolution of Haemosporidia Lineages**

All lineages returned by the PHASE (Stephens *et al*. 2001) software output were compared against the top three matches in both GenBank (Dennis *et al.* 2005) and the MalAvi Database (Bensch *et al.* 2009) (Table S3). For all lineages appearing only once in the dataset, either as a single unique infection in a host (n=8), or as a one unique sequence part of a double infection (n-=5), we verified these sequences via inspection of both forward and reverse eletropherograms. For unique single infections, sequences were unambiguous and easily verified. For double infections, we looked for evidence of each of the base pairs recovered via computational methods at the base positions where double peaks occurred in the eletropherograms. Caution should be taken when double infections are resolved and found to contain two unique lineages found nowhere else in the dataset. For our unique sequences appearing as part of a double infection, in all cases expect one (in *Glyphorynchus spirurus*, a rare host in our sampling), these lineages were co-infected with a common lineage found in at least 1 other individual sampled, suggesting double infections most often involve an infection of a common lineage with one that is rare. For our single exception, phase probabilities were low at three base pairs in this double infection, and caution should be taken in interpreting these two lineages (see Results).

**Measures of Ecological Gradients**

We extracted the fifty-year mean climatologies for each of 19 environmental variables from the WorldClim database ([www.worldclim.org](http://www.worldclim.org/) (Hijmans et al. 2005)) as a surrogate of ecological differences between sites. These variables capture numerous aspects of both precipitation and temperature and are available at 1-km resolution for our area of study. In addition to climate variables, we extracted the elevation at each study site at 30 arc second resolution (= 1 km at sites close to the equator) using data collected from the Shuttle Radar Topography Mission (SRTM, USGS/NASA-NGA 2004). To account for any spatial autocorrelation at sites or across sites, we included latitude and longitude as predictor variables in all model runs and evaluations.

**Tree Regressions and Random Forests**

We determined the ecological factors (measured by climate, elevation, and geography) that best explained differences in haemosporidia prevalence across our study sites using tree regression as implemented in the *tree* package (Ripley 1996) and random forests as implemented in *randomForest* (Liaw & Wiener 2002), both operating within the R statistical framework (R Foundation for Statistical Computing 2011). Tree regression is a method of non-linear recursive bifurcating regression by which response variables are split according to measures of deviance (a measure similar to variance), by each of the predictor variables. The best predictor variables are those that bifurcate the response variable (in this case the prevalence of haemosporidia) such that deviance in each split is minimized. This process is repeated until splits no longer lead to statistically significant differences between resulting groups. Random forests represent numerous tree regressions whereby iterations represent random subsets of both predictors and response records such that predictors can be evaluated given random inclusion of both other predictors in the model, as well as new data in the form of records withheld for testing (Breiman 2001).

We ran tree regression using default settings in the package *tree*. For random forests, we ran 5000 trees with 19 predictor variables and included variable importance in all model evaluations. Variable importance scores are reported in Fig. S3. Total percent variation reported in our analyses represents the OOB (or “out-of-bag”) variation explained by each model; this is variation explained in new test data that the model never saw during training (Breiman 2001).

**Construction of Host Phylogeny**

Alignments were carried out using the MAFFT software (Katoh et al. 2002) implemented in Geneious (Drummond et al. 2011). We used PartitionFinder (Lanfear et al. 2012) to choose both a partitioning strategy and models of molecular evolution, and the optimal partitioning strategy selected via PartitionFinder was a two-partition model with first and 3^rd^ codon positions combined and assigned the GTR+G model of molecular evolution, and 2^nd^ positions assigned the HKY+G model. We performed Bayesian phylogenetic analyses using MrBayes v3.2 (Huelsenbeck and Ronquist 2001; Ronquist et al. 2012) for the concatenated data set, and each locus individually, and MrBayes analyses were carried out through the CIPRES Web portal ([www.phylo.org](http://www.phylo.org/)). Bayesian analyses consisted of two independent runs each comprising four incrementally heated chains that ran for 10,000,000 generations. We sampled the posterior distribution every 1000 generations, and checked for stationarity by ensuring that the potential scale reduction factor equaled 1, and the average standard deviation of split frequencies between independent runs approached 0. We examined the MCMC samples in Tracer and AWTY (Rambaut and Drummond 2007; Nylander et al. 2008) to ensure that all chains were sampling from the same target distribution, and we discarded the first 25% of samples as burn-in, provided the chains had reached stationarity prior to this point. Final tree was rooted using a Galliform (*Anas platyrhynchos*) that was excluded for taxonomic distance measurements (Fig. S4).

**
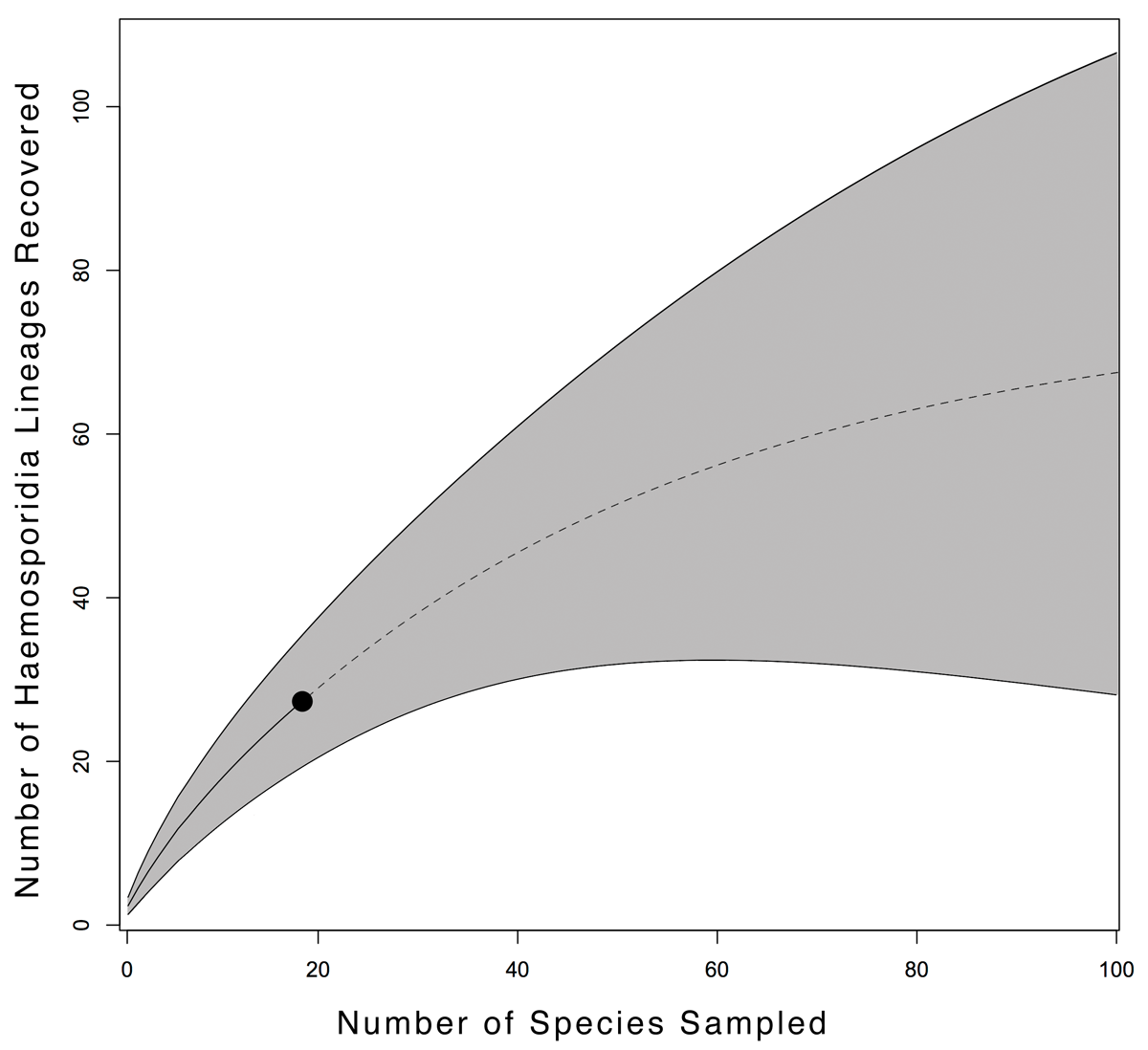
**

Figure S1. Rarefication curve showing the increase in number of discovered lineages of avian haemosporidia as number of species sampled (at random) increase. Greyed area represents upper and lower 95% confidence intervals. We detected a total of 28 unique lineages (filled circle) including 5 members of *Leucocytozoon*) in our data set in 19 host species. Extrapolation given our current discovery rate (dashed line) suggests over 100 lineages could exist provided at least 100 host species were sampled. The Northern Andes are home to over 2000 species of potential avian hosts (Herzog and Kattan 2011).


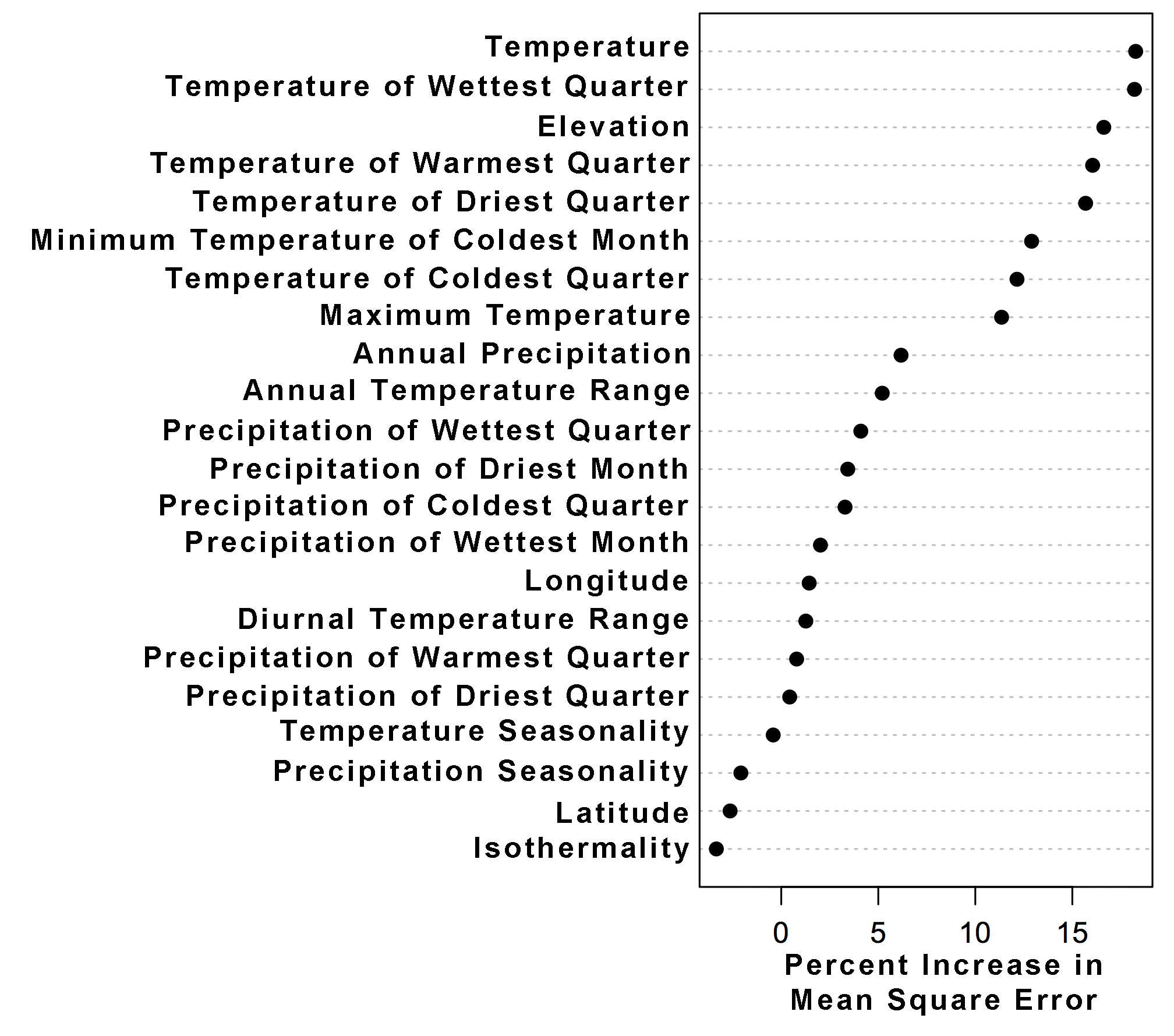


Figure S2. Variable importance scores for all ecological variables and including geographic variable (latitiude and longitude) used as predictors to explain avian haemosporidia pravelence in random forest models. The larger the percent mean square error introduced when removing a variable, the more important that variable is deemed in the full model. Temperature (correlated measures of annual mean temperature and mean temperature of the wettest quarter) and elevation ranked among the top most important variables in predicting haemosporidia prevalence in the Northern Andes. All variables represent means unless otherwise stated.


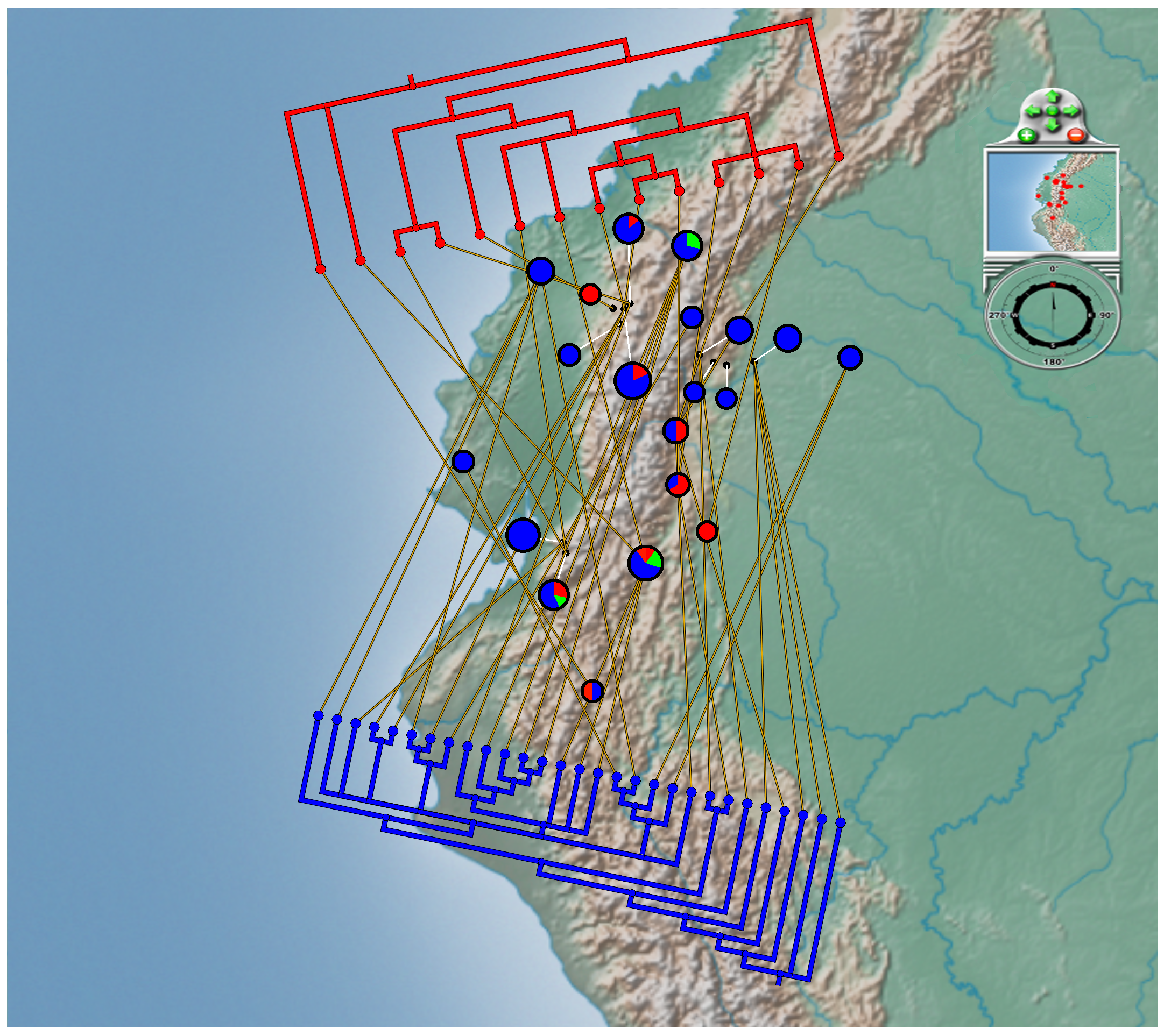


Figure S3. Optimal angles of *Haemoproteus* (blue) and *Plasmodium* (red) lineages along linear axes with respect to reconstructed maximum likelihood trees. For *Haemoproteus*, the least number of crossings occurred at an angle of 243.1°, resulting in 59 crossings. While not crossing significantly fewer than 10,000 random topologies, this angle resulted in the fewest crossings (59) for the recovered phylogeny. An optimal angle of 101.3° for the *Plasmodium* phylogeny resulted in nine crossings, significantly fewer (*p* < 0.05) than 10,000 random topologies.

**
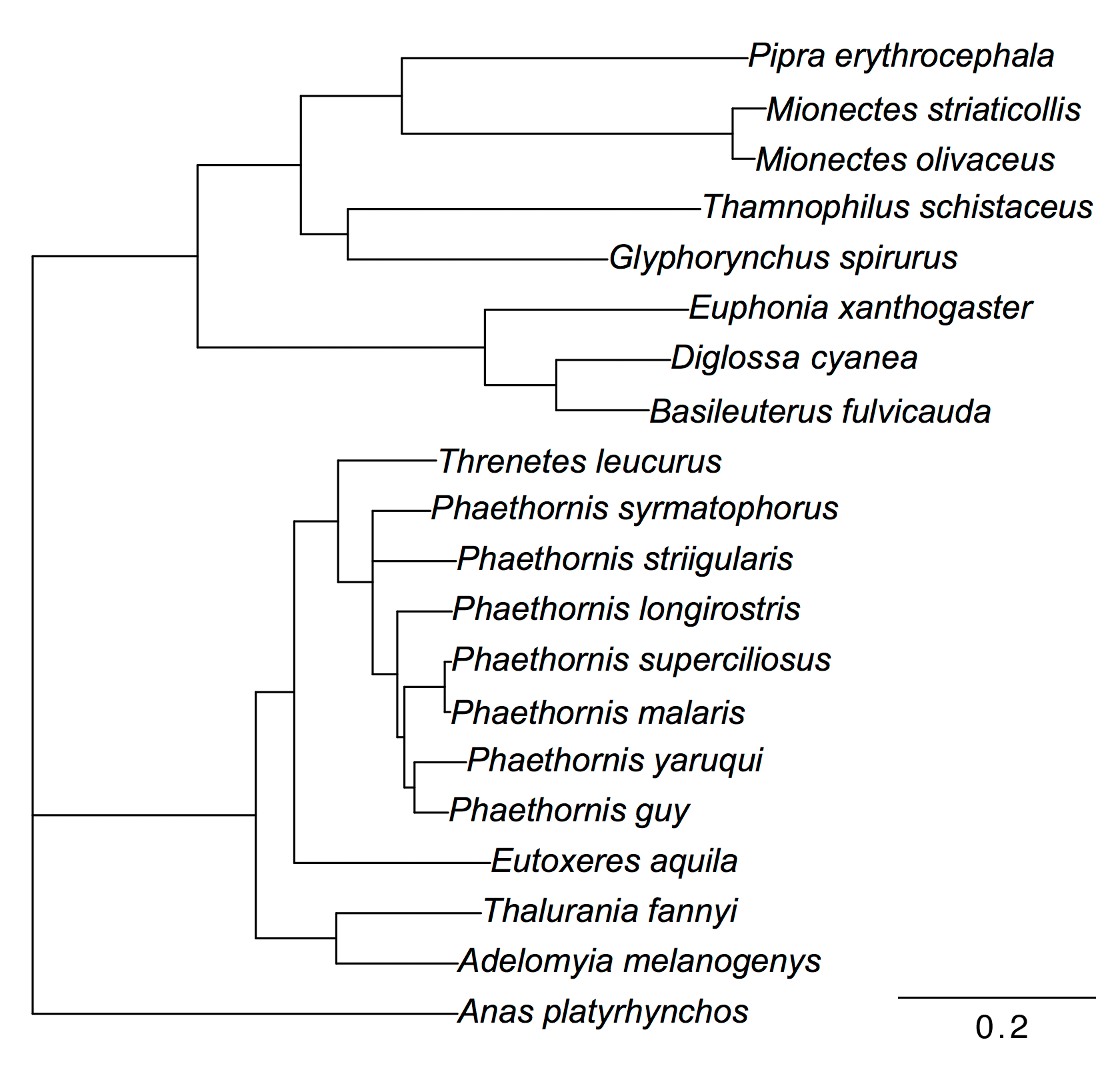
**

Figure S4. Phylogeny of host species found to be infected with avian haemosporidia in Ecuador. Tree was constructed using the full mitochondrial dehydrogenase subunit 2 (ND2) that was 1041 base pairs in length. Sequences for all species were downloaded from GenBank, including an outgroup within Galliformes (*Anas platyrhynchos*) that was excluded in pairwise host taxonomic distances.

Table S1. Site locations, prevalence, and number of lineages of avian haemosporidia at sites within the study region. Temperature values represent annual mean temperature at each site. Prevalence was not calculated for sites where the number of sampled individuals was 10 or less. Confidence Intervals (95%) are presented next to prevalence values as an estimate of uncertainty.

Table S2. Diversity indices of lineages of avian haemosporidia in the study region. Asterisks indicate those lineages that had significantly lower phylogenetic diversity of hosts as compared to 999 iterations of random host structure and infection (See Methods). Grayed boxes indicates lineages infecting multiple hosts, suggesting *Plasmodium* parasites are more generalist in the region as compared to lineages of *Haemoproteus*.

Table S3. Lineages recovered and closest matches in GenBank and MalAviDatabase. A total of 7 *Plasmodium*, 16 *Haemoproteus*, and 5 *Leucocytozoon* were found across the study region. Across these lineages, only 8 (2 *Plasmodium*, 6 *Haemoproteus*) matched 100% to previously recovered avian haemosporidia sequences.

Table S4. Double infection resolved using PHASE software. Lineages found in each mixed infection are listed (Table S3).

**References**

Bensch S., O. Hellgren, and J. Perez-Tris. 2009. MalAvi: a public database of malaria parasites and related haemosporidians in avian hosts based on mitochondrial cytochrome b lineages. *Molecular Ecology Resources* **9**:1353–1358.

Breiman L. 2001. Random Forests. *Machine Learning* **45**:5-32.

Chaorattanakawee S., O. Natalang, H. Hananantachai, M. Nacher, A. Brockman, S. Krudsood, S.

Looareesuwan, and J. Patarapotikul. 2003. Storage duration and polymerase chain

reaction detection of *Plasmodium falciparum* from blood spots on filter paper. *The*

*American Journal of Tropical Medicine and Hygiene* **69**:42-44.

Dennis A. B., I. Karsch-Mizrachi, J. L. David, O. James, and L.W. David. 2005. GenBank. *Nucleic Acids Research* **1**:D34-D35.

Drummond, A. J., B. Ashton, S. Buxton, M. Cheung, A. Cooper, C. Duran, M. Field, J. Heled, M. Kearse, S. Markowitz, R. Moir, S. Stones-Havas, S. Sturrock, T. Thierer, and A. Wilson. 2011. Geneious v5.4, <http://www.geneious.com/>.

Herzog S. K., and G. H. Kattan. 2011. Patterns of diversity and endemism in the birds of the tropical Andes. In *Climate change and biodiversity in the tropical Andes*, edited by S. K. Herzog, R. Martínez, P. M. Jørgensen, and H. Tiessen. Inter-American Institute for Global Change Research (IAI) and Scientific Committee on Problems of the Environment (SCOPE).

Hijmans R.J. , S. E. Cameron, J. L. Parra, P. G. Jones, and A. Jarvis. 2005. Very high resolution interpolated climate surfaces for global land areas. *International Journal of Climatology* **25**:1965-1978.

Huelsenbeck, J. P., and F. Ronquist. 2001. MRBAYES: Bayesian inference of phylogeny. *Bioinformatics* **17**:754–755.

Katoh K., K. Misawa, K. Kuma, and T. Miyata T. 2002. MAFFT: a novel method for rapid multiple sequence alignment based on fast Fourier transform. *Nucleic Acids Research* **30**:3059-3066.

Lanfear R., B. Calcott, S. Y. W. Ho, and S. Guindon. 2012. PartitionFinder: combined selection of partitioning schemes and substitution models for phylogenetic analyses. *Molecular Biology & Evolution* **29**:1695-1701.

Liaw A., and M. Wiener. 2002. Classification and regression by randomForest. *R News* **2**:18–22.

Nylander J. A., J. C. Wilgenbusch, D. L. Warren, and D. L. Swofford DL. 2008. AWTY (Are We There Yet): a system for graphical exploration of MCMC convergence in Bayesian phylogenetics. *Bioinformatics* **24**:581-583.

R Foundation for Statistical Computing. 2011. R: A Languauge and Environment for Statistical Computing, Reference Index Version 2.13.1. R Foundation for Statistical Computing, Vienna, Austria.

Rambaut A., and A. J. Drummond. 2007. Tracer v1.4, <http://beast.bio.ed.ac.uk/Tracer>.

Richardson A. J., N. Narendran, R. H. Guymer, H. Vu, and P. L. Baird. 2006. Blood storage at 4° C- factors involved in DNA yield and quality. *Journal of Laboratory Clinical Medicine* **147**:290-294.

Ripley, B. D. 1996. *Pattern recognition and neural networks*. Cambridge; New York: Cambridge University Press.

Ronquist F., M. Teslenko, P. van der Mark, D. L. Ayres, A. Darling, S. Höhna, B. Larget, L. Liu, M. A. Suchard, and J. P. Huelsenbeck. 2012. MRBAYES 3.2: Efficient Bayesian phylogenetic inference and model selection across a large model space. *Systematic Biology* **61**:539-542.

United States Geological Survey. 2004. Shuttle Radar Topography Mission, 30 Arc Second Scene SRTM. Global Land Cover Facility. University of Maryland, College Park, Maryland.

Valkiūnas G., T. A. Iezhova, C. Loiseau, R. N. M. Sehgal. 2009. Nested cytochrome *B* polymerase chain reaction diagnostics detect sporozoites of haemosporidian parasites in peripheral blood of naturally infected birds. *Journal of Parasitology* **95**:1512-1515.

Waldenström J., S. Bensch, D. Hasselquist , and Ö. Östman. 2004. A new nested polymerase chain reaction method very efficient in detecting *Plasmodium* and *Haemoproteus* infections from avian blood. *Journal of Parasitology* **90**:191–194.
